# Kinderarmut in Deutschland: Entstehungsursachen und Gegenmaßnahmen

**DOI:** 10.1007/s12054-020-00344-w

**Published:** 2020-12-17

**Authors:** Christoph Butterwegge

**Affiliations:** Universität zu Köln, Köln, Deutschland

**Keywords:** Kinderarmut, Mütterarmut, Familienarmut, Armutsbekämpfung, Kinder- und Jugendhilfe, Mindestlohn, Ungleichheit

## Abstract

Bei der Kinderarmut handelt es sich um eine der verheerendsten Ausprägungen des Polarisierungsprozesses, der die sozioökonomische Ungleichheit als das eigentliche Kardinalproblem der Bundesrepublik, wenn nicht der ganzen Menschheit, verschärft hat. Daher muss Armutsbekämpfung auf sämtlichen Ebenen des föderalen Systems (Bund, Länder und Kommunen) sowie allen dafür geeigneten Politikfeldern ansetzen, von denen im Artikel exemplarisch die Arbeitsmarkt‑, Beschäftigungs- und Sozialpolitik, die Familienpolitik, die Bildungs- und Schulpolitik sowie der Wohnungs‑, Wohnungsbau- und Stadtentwicklungspolitik behandelt werden.

Kaum noch jemand leugnet, dass die Armutsbetroffenheit von Kindern in der Bundesrepublik viel zu hoch ist. Wenn in einem so reichen Land mehr als jedes fünfte Kind in einer sozial benachteiligten Familie aufwächst, muss von einem handfesten Skandal gesprochen werden. Bei der Kinderarmut, die zuerst totgeschwiegen, später verharmlost und wieder verdrängt wurde, handelt es sich um eine der verheerendsten Ausprägungen des Polarisierungsprozesses, der die sozioökonomische Ungleichheit als das eigentliche Kardinalproblem der Bundesrepublik, wenn nicht der ganzen Menschheit, verschärft hat.

Kinderarmut ist ein viel zu ernstes Problem, um seine Lösung den unmittelbar betroffenen Familien sowie meistenteils gleichfalls hilflosen Erzieher_innen, Lehrer_innen und Sozialarbeiter_innen zu überlassen. Nur durch sozialstaatliche Intervention kann sie verringert und ihre Neuentstehung verhindert werden.

## Wurzeln der Armutsentwicklung

Kinderarmut beweist, dass sich Armutsbetroffenheit in aller Regel nicht mit individuellem (Fehl‑)Verhalten, sondern nur mit den gesellschaftlichen Verhältnissen erklären lässt, von denen Menschen abhängig sind. Maksim Hübenthal (2018) hat am Beispiel der Plenardebatten im Bundestag zu diesem Themenbereich vier Konstruktionsweisen herausgearbeitet, die für ihn genuine Erklärungsansätze repräsentieren: die Konstruktion von Kinderarmut als

- Erziehungsarmut,
- Bildungsarmut,
- Geldarmut und als
- Rechtearmut.

Diesen Sinngebungsvarianten entsprechen seiner Meinung nach unterschiedliche Gegenstrategien:

- Für die Vertreter_innen der Erziehungsarmutskonstruktion stehen Maßnahmen der „aktivierenden Arbeitsmarktpolitik“ und zur „Reeducation“ der betroffenen Familien im Vordergrund, etwa der Ausbau von Kindertagesstätten zu Eltern-Kind-Zentren, in denen auf „Problemfamilien“ eingewirkt werden soll;
- bei Anhänger_innen der Bildungsarmutskonstruktion sind es Maßnahmen im Bereich von Kindertageseinrichtungen und Schulen, mit denen die Bildungschancen verbessert werden sollen;
- bei Repräsentant_innen der Geldarmutskonstruktion dominieren Maßnahmen zur Umverteilung sowie zur materiellen Besserstellung von Eltern im Niedriglohnsektor und im Transferleistungsbezug;
- die Vertreter_innen der Rechtearmutskonstruktion favorisieren Maßnahmen zur Aufwertung der Rechtsposition von Kindern, etwa die Verankerung besonderer Kinderrechte in der Verfassung.

Obwohl Hübenthal (ebd., S. 243) zu dem Schluss gelangt, „dass einzig die Rechtearmutskonstruktion mit ihrem generational angelegten Verständnis sozialer Gerechtigkeit in der Lage ist, die Problemlagen von Kindern als Kinder zu erfassen, diese Bevölkerungsgruppe umfassend in den Blick zu nehmen sowie den Vergleich zu den Erwachsenen zu ermöglichen“, scheinen Zweifel angebracht, ob die Fixierung auf diese oder eine andere Kinderarmutskonstruktion sinnvoll ist. Das deutsche System der sozialen Sicherung ist zwar erwerbsarbeits-, ehe- und *erwachsenen*zentriert, die einseitige Konzentration auf *Kinder*rechte greift aber viel zu kurz, wenn es um die Bekämpfung von Armut und sozialer Ausgrenzung geht. Schließlich sind Geld und Recht zwei Medien, über die sozioökonomische Ungleichheit in der bürgerlich-kapitalistischen Gesellschaft abgesichert wird, während Bildung und Erziehung dazu beitragen, sie zu reproduzieren und zu legitimieren. Und wie soll die von Hübenthal augenscheinlich präferierte „kindergerechte Generationengesellschaft“ entstehen, wenn die Ausbeutungsmechanismen, Eigentumsverhältnisse und Verteilungsschieflagen des Finanzmarktkapitalismus unangetastet bleiben?

Maßgeblich für die Zunahme der Kinderarmut in den vergangenen Jahren und Jahrzehnten waren gesellschaftliche Entwicklungsprozesse, die von einer Deregulierung des Arbeitsmarktes über die Pluralisierung der Familienformen bis zu einer von Kritiker_innen und seinen Hauptnutznießer_innen als systematische Demontage erlebten Restrukturierung des Sozialstaates (vgl. hierzu: Butterwegge 2018, S. 113 ff.) reichen. Die sich aufgrund einer sozialen Polarisierung im Gefolge der neoliberalen Globalisierung vertiefende Kluft zwischen Arm und Reich (vgl. hierzu: Butterwegge et al. 2008, S. 48 ff.; Butterwegge 2020a, S. 254 ff.) hat gerade das Leben junger Menschen entscheidend geprägt.

Kinder und Jugendliche gehörten auch zu den Hauptleidtragenden der Covid-19-Pandemie. Kita- und Schulschließungen, die in höheren Jahrgangsstufen größtenteils mit einer beschleunigten Digitalisierung des Unterrichts (Homeschooling und E‑Learning) einhergingen, haben die Benachteiligung von Kindern aus finanzschwachen Familien im Bildungsbereich verstärkt (vgl. hierzu: Butterwegge 2020b, S. 161 ff.). Häufig standen ihnen keine geeigneten digitalen Endgeräte (PC, Laptop oder Tablet und Drucker) im Haushalt zur Verfügung, die nötig gewesen wären, um den Kontakt zur Schule zu halten und nicht ins Hintertreffen gegenüber materiell bessergestellten Klassenkamerad_innen zu geraten. Michael Klundt (2020) nennt die Coronakrise einen „Armutskatalysator“ für die Familien, weil eine Privatisierung der Krisenrisiken stattgefunden habe und von einem kindergerechten Krisenmanagement durch die politisch Verantwortlichen keine Rede sein könne. Vielmehr drohe ein gesellschaftlicher Rückschritt und aus der Bundesrepublik ein „kinderfeindliches Land“ zu werden.

## Armutsbekämpfung auf mehreren Politikfeldern

Auf welchen Politikfeldern, mit welcher Strategie und mit welchen Mitteln man die Armut von Kindern zu bekämpfen sucht, hängt primär davon ab, welche Ursache(n) man für das Phänomen verantwortlich macht. Wenn die Entstehung von (Kinder‑)Armut nicht monokausal zu begreifen ist, sondern multiple, teilweise eng miteinander verknüpfte Ursachen hat, ist sie auch nur mehrdimensional zu bekämpfen. Michael Klundt (2019, S. 165) fordert daher ein „mehrdimensionales Anti-Kinderarmuts-Konzept“, das mit einem grundlegenden Wandel der Regierungspolitik verbunden sein müsse, die oft genug bloß soziale „Trostpflästerchen“ für strukturelle Probleme wie die wachsende Ungleichheit biete.

Da die Kinderarmut öffentlich beklagt, aber nicht energisch bekämpft wird, sollte zunächst ein politisches Klima geschaffen werden, das ihre „strukturelle Unsichtbarkeit“ beendet, von der Daniel März (2017, S. 14) spricht: „Kindheit in Armut kann sich zwar des Mitgefühls ihrer gesellschaftlichen Umwelt gewiss sein, jedoch nicht eines hinreichenden gesamtgesellschaftlichen Veränderungswillens.“ Nötig sind mehr Sensibilität für Prekarisierungs‑, Marginalisierungs- bzw. Pauperisierungsprozesse sowie eine höhere Sozialmoral, die aufgrund der Wohnungsnot und des Mietwuchers in Großstädten und Ballungsgebieten der Bundesrepublik allmählich bis in die Mittelschicht reichende Desintegrations‑, Exklusions- und Deprivationstendenzen als Gefahr für den gesellschaftlichen Zusammenhalt begreift.

## Arbeitsmarkt-, beschäftigungs- und sozialpolitische Maßnahmen

Eine konsequente Beschäftigungspolitik würde nicht nur die Arbeitslosigkeit verringern, sondern auch der Familien- und Kinderarmut nachhaltig entgegenwirken. Sie müsste von einer Umverteilung der Arbeit durch den Abbau von Überstunden (mittels eines Verbots *bezahlter* Überstunden) und die sukzessive Verkürzung der Wochenarbeitszeit (bei einem Personal- und Lohnausgleich zumindest für Geringverdiener_innen) wie der Lebensarbeitszeit über Zukunftsinvestitionsprogramme des Bundes und der Länder bis zur Schaffung eines öffentlich geförderten Dienstleistungssektors alle Möglichkeiten der wirtschaftspolitischen Interventionstätigkeit für die Schaffung von mehr Stellen nutzen.

Armutsverschärfend wirkt, „dass in Deutschland kein flächendeckendes Netz von tariflichen Mindeststandards zur Einkommensfestsetzung mehr existiert, auch wenn sich ein Teil der nicht an Tarifverträge gebundenen Arbeitgeber an den bestehenden Branchentarifverträgen orientiert.“ (Bispinck und Schäfer 2006, S. 271) Da die Erosion des Normalarbeitsverhältnisses maßgeblich zur Verbreitung von (Kinder‑)Armut beiträgt, ist die Festigung des Flächentarifvertrages, der besonders in Ostdeutschland kaum noch Breitenwirkung entfaltet, ein weiteres Element ihrer wirkungsvollen und nachhaltigen Bekämpfung. Minijobs müssen in sozialversicherungspflichtige Beschäftigungsverhältnisse umgewandelt, Leiharbeitsverhältnisse entweder ganz verboten oder wieder stärker reguliert werden.

Aus dem Umstand, dass die Armut nicht mehr nur Erwerbslose trifft, sondern in Teilbereiche der Arbeit vorgedrungen ist, haben CDU, CSU und SPD nach langem Zögern die Konsequenz eines gesetzlichen Mindestlohns gezogen, der zum 1. Januar 2021 auf 9,50 € erhöht wurde. Zwar scheint der großkoalitionäre Mindestlohn die Massenkaufkraft aufgrund deutlicher Lohnerhöhungen gestärkt und die Binnenkonjunktur belebt zu haben, weniger erfolgreich war er allerdings bei der Armutsbekämpfung. So ging die Anzahl der sog. Hartz-IV-Aufstocker_innen bloß um 1,1 Prozentpunkte zurück (vgl. Pusch 2018, S. 3 f.), weil Geringverdiener_innen mit Kindern, die in einer Großstadt mit den heute üblichen hohen Mieten wohnen, keine Chance hatten, der Transferabhängigkeit durch Anhebung ihres Lohns auf die gesetzlich vorgeschriebene Mindesthöhe zu entkommen.

Nur ein Mindestlohn in existenzsichernder Höhe, die Streichung sämtlicher (vulnerable Gruppen wie Langzeitarbeitslose, Jugendliche ohne Berufsabschluss und Kurzzeitpraktikanten treffender) Ausnahmen sowie eine flächendeckende Überwachung durch die Finanzkontrolle Schwarzarbeit des Zolls könnten bewirken, dass der Mindestlohn überall ankommt. „Gleichzeitig müssen die Möglichkeiten von Beschäftigten, ihre Mindestlohnansprüche gegenüber den Unternehmen durchzusetzen, gestärkt werden.“ (Schulten und Weinkopf 2015, S. 90) Erwägenswert sind ein Verbandsklagerecht (für die Gewerkschaften) und eine Kontrollinstitution nach britischem Vorbild.

## Familienpolitik

Kinderarmut lässt sich in der Regel auf Frauen- bzw. Mütterarmut zurückführen, sodass ein Hebel zu ihrer Verringerung in einer Erhöhung der weiblichen Erwerbsbeteiligung liegt, was eine nachhaltige Verbesserung der Vereinbarkeit von Familienarbeit und Berufstätigkeit durch Schaffung von mehr (Teilzeit‑)Stellen einerseits sowie mehr öffentlichen Kinderbetreuungseinrichtungen, die kostengünstiger bzw. beitragsfrei zur Verfügung gestellt werden müssten, andererseits voraussetzt. Nötig wäre darüber hinaus eine (gesetzlich zu regelnde) Rückbindung der Arbeit selbst wie der Arbeitszeitregelungen in Betrieben und öffentlichen Verwaltungen an die Lebensbedürfnisse der Beschäftigten und ihrer Familien.

Dies würde eine völlige Neujustierung des Normalarbeitsverhältnisses erfordern: Beschäftigte müssten im Laufe ihres Lebens zwischen Vollzeit‑, Teilzeitarbeit und Arbeitsunterbrechung ohne Verluste an sozialer Sicherung und Weiterbildungsmöglichkeiten wechseln können, und Arbeitgeber sowohl in der Arbeitszeitgestaltung wie auch beim Arbeitsvolumen auf die unterschiedlichen, im Lebensverlauf wechselnden Interessen der Beschäftigten mehr Rücksicht nehmen (vgl. Stolz-Willig 2003, S. 221). Die von CDU, CSU und SPD im Januar 2019 eingeführte „Brückenteilzeit“ gilt nur in Betrieben mit in der Regel über 45 Beschäftigten und räumt dem Arbeitgeber das Recht ein, den Wunsch nach Verringerung der Arbeitszeit abzulehnen, soweit ihm betriebliche Gründe entgegenstehen.

Überfällig ist eine Neuordnung des Familienleistungsausgleichs, der die folgenden Kriterien erfüllen müsste, um dem Ziel einer wirksamen Bekämpfung bzw. Vermeidung von Kinderarmut dienen zu können:

- Transferleistungen und steuerliche Freistellungen müssten sich an einem einheitlichen soziokulturellen Mindestbedarf für Kinder orientieren;
- sie dürften nicht zu unterschiedlichen Entlastungs- und Unterstützungsleistungen führen, also Familien mit niedrigeren Einkommen benachteiligen;
- um die Verarmung von Familien auszuschließen, bedarf es eines armutsfesten, nichtdiskriminierenden bzw. -stigmatisierenden und repressionsfreien Transfersystems mit sachgerecht ermittelten und deutlich höheren Regelbedarfen, als es sie bei Hartz IV gibt (vgl. Zander 2000, S. 100 f.; Butterwegge 2018, S. 415 f.).

Finanztransfers, die der Staat an Familien zahlt, haben für manchen Kritiker den Nachteil, dass sie ausgerechnet die am meisten von Armut betroffenen Kinder nicht immer erreichen, weil das den Empfänger_innen an allen Ecken und Enden fehlende Geld womöglich für anderes ausgegeben wird. Umso zweckmäßiger wäre es, wenn gerade benachteiligte oder „abgehängte“ Regionen befähigt würden, ihre soziale, Bildungs- und Betreuungsinfrastruktur so weit zu entwickeln, dass die dort extrem hohe Kinderarmut sinkt. Nur wenn genügend Kindertagesstätten, gut ausgestattete Schulen und ausreichend Freizeitangebote (vom öffentlichen Hallenbad über den Jugendtreff und das Museum bis zum Tierpark) vorhanden sind, kann verhindert werden, dass ein Großteil der nachwachsenden Generation unterversorgt und perspektivlos bleibt.

## Bildungs- und Schulpolitik

Bildung spielt eine wichtige Rolle, wenn es um bessere Aufstiegschancen für junge Menschen aus sozial benachteiligten Familien geht, wiewohl sie nicht verabsolutiert und zum gesamtgesellschaftlichen Wundermittel im Kampf gegen die Kinderarmut stilisiert werden darf (vgl. Butterwegge 2019). Ganztagsschulen, die (beitragsfrei zur Verfügung gestellte) Kindergarten‑, Krippen- und Hortplätze ergänzen sollten, haben einen Doppeleffekt: Einerseits werden von Armut betroffene oder bedrohte Kinder umfassender betreut und systematischer gefördert, andererseits können ihre Eltern leichter als sonst einer Vollzeitbeschäftigung nachgehen, was sie finanzielle Probleme eher meistern lässt. Durch die Ganztags- als Regelschule lassen sich soziale Benachteiligungen insofern kompensieren, als eine bessere Versorgung der Kinder mit Nahrung (gemeinsame Einnahme des Mittagessens), eine gezielte Förderung leistungsschwächerer Schüler_innen etwa bei der Erledigung von Hausaufgaben und eine sinnvollere Gestaltung der nachmittäglichen Freizeit erfolgen würden.

Wer von der Gesamt- bzw. Gemeinschaftsschule, die Kinder aller Bevölkerungsschichten länger gemeinsam unterrichtet, nicht sprechen will, sollte auch von der Ganztagsschule schweigen. Letztere degeneriert zur bloßen Verwahranstalt, wenn sie nicht in ein bildungspolitisches Alternativkonzept integriert wird, das bemüht ist, den Bildungserfolg von der sozialen Herkunft zu entkoppeln. In „einer Schule für alle“ nach skandinavischem Vorbild wäre kein Platz für die frühzeitige Aussonderung „leistungsschwacher“ oder „bildungsferner“ Kinder, die arm sind bzw. aus sog. Problemfamilien stammen. Mit einer inklusiven Pädagogik, die keine „Sonderbehandlung“ für bestimmte Gruppen mehr kennt (vgl. dazu: Reich 2014; Ottersbach et al. 2016), könnte man sozialer Desintegration und damit dem Zerfall der Gesellschaft insgesamt entgegenwirken.

Nötig wäre eine sehr viel intensivere Zusammenarbeit bzw. eine stärkere Verzahnung von Schule und Jugendhilfe, als sie bisher besteht (vgl. dazu: Henschel et al. 2009). Chassé et al. (2007, S. 342) formulieren eine doppelte Aufgabe: „Öffnungen der Schule gegenüber dem Stadtteil bzw. dem Freizeitbereich könnten einerseits zu einer gemeinwesenorientierten Schule führen. Auf der anderen Seite müssten die Institutionen der Kinder- und Jugendhilfe – sicherlich oft in Kooperation mit den Schulen, vor allem im Kontext von Ganztagsschulen – lebensweltnahe attraktive Freizeit‑, Förder- und Bildungsangebote entwickeln, mit denen die Kinder erreicht werden können, die von herkömmlichen Vereinen und kommerziellen Angeboten keinen Gebrauch machen können.“ Sinnvoll ist nach Überzeugung der Verfasser_innen „eine Neubestimmung des Verhältnisses von Bildung und Jugendhilfe“ (ebd., S. 343), die sich an einer übergreifenden Integrationsperspektive orientieren muss.

## Wohnungs‑, Wohnungsbau- und Stadtentwicklungspolitik

Aufgrund der zunehmenden Wohnungsnot und des Mietwuchers in Groß- bzw. Universitätsstädten sowie Ballungszentren finden heute selbst Normalverdiener_innen oft keine bezahlbare Mietwohnung mehr. Während der Wohnungsmarkt dereguliert wurde, erfasste den sozialen Wohnungsbau eine gleichfalls politisch herbeigeführte Schwindsucht: Momentan fallen jährlich viermal so viele Wohnungen aus der Belegungsbindung heraus, wie neu hinzukommen.

Lange bevor Wohnungsmangel und Mietwucher als „Neue Soziale Frage“ bzw. als eine zentrale politische Herausforderung des 21. Jahrhunderts entdeckt und zum Thema in den Massenmedien wurde, gelangte die Fuldaer Soziologin Monika Alisch (2009, S. 51) zu dem Schluss, dass sich die Verschärfung der (Kinder‑)Armut nicht zuletzt als „Ergebnis stadtentwicklungspolitischer Entscheidungen“ darstellt. Das im *Baugesetzbuch* verankerte Politikfeld der „sozialen Stadtentwicklung“ gewinnt daher zunehmend an Bedeutung. „Soziale Stadtentwicklung knüpft an die These an, dass Städte nur eine Chance haben, die Auswirkungen ökonomischer Umstrukturierung sozialverträglich zu beantworten, wenn sie einen sozialen Ausgleich anstreben.“ (Alisch 2001, S. 52)

Ob eine Bund-Länder-Offensive, wie sie das 1999 aufgelegte und zuletzt ausgeweitete Gemeinschaftsprogramm „Stadtteile mit besonderem Entwicklungsbedarf – die Soziale Stadt“ darstellt, geeignet ist, strukturelle Probleme nach Art der sozialen Benachteiligung bzw. Verarmung von Alleinerziehenden und kinderreichen Familien zu lösen oder zu mildern, bleibt umstritten (vgl. Walther 2002; Greiffenhagen und Neller 2005; Hanesch 2011). Zumindest bedarf es einer Anerkennung der Erledigung solcher Aufgaben als Regelleistung, denn Soziale Arbeit kann nicht als Feuerwehr fungieren und muss unabhängig von familien- und sozialpolitischen Konjunkturen einerseits sowie parlamentarischen Mehrheiten andererseits garantiert sein.

Ohne eine grundlegende Wende in der Wohnungspolitik wird die Obdachlosigkeit in Deutschland weiter zunehmen. Da sich Räumungsklagen und Zwangsräumungen mehren, ist die Verankerung eines „Grundrechts auf Wohnraum“ in der Verfassung überfällig, für das der heutige Bundespräsident Frank-Walter Steinmeier in seiner Doktorarbeit plädiert hat. Staat und Behörden müssten, forderte Steinmeier (1992, S. 394) im Grundgesetz „zum Bau und Erhalt preisgünstigen Wohnraums für breite Bevölkerungskreise“ verpflichtet werden, und es dürfe, so Steinmeier (ebd., S. 395) weiter, keine Wohnung z. B. wegen aufgelaufener Mietschulden geräumt werden, bevor nicht „zumutbarer Ersatzwohnraum“ zur Verfügung stehe.

## Fazit

Armutsbekämpfung sollte auf sämtlichen Ebenen des föderalen Systems (Bund, Länder und Kommunen) sowie allen dafür geeigneten Politikfeldern ansetzen, wiewohl nur ausgewählte behandelt werden konnten. Bloß durch eine konzertierte Aktion im Bereich der Wirtschafts‑, Steuer- und Finanzpolitik, der Arbeitsmarkt- und Beschäftigungspolitik, der Sozial- und Gesundheitspolitik, der Familienpolitik, der Bildungspolitik sowie der Wohnungs‑, Wohnungsbau- und Stadtentwicklungspolitik sind dauerhafte Erfolge möglich. Dabei müssen auch Landes- und Kommunalpolitik mehr Verantwortung übernehmen (vgl. dazu: Gintzel et al. 2008; Lutz 2010). Je umfassender die Maßnahmen zur Verringerung bestehender und/oder zur Verhinderung der Entstehung neuer Kinderarmut angelegt und je besser sie aufeinander abgestimmt sind, desto eher ist dem Problem beizukommen.

## References

[1] Alisch M, Alisch M (2001). Stadtteilmanagement. Zwischen politischer Strategie und Beruhigungsmittel. Stadtteilmanagement. Voraussetzungen und Chancen für die soziale Stadt.

[2] Alisch M, Lutz R, Hammer V (2009). Der Einfluss von Stadtentwicklungs- und Wohnungspolitik auf Armut. Wege aus der Kinderarmut. Gesellschaftspolitische Rahmenbedingungen und sozialpädagogische Handlungsansätze.

[3] Bispinck R, Schäfer C, Schulten T, Bispinck R, Schäfer C (2006). Niedriglöhne und Mindesteinkommen: Daten und Diskussionen in Deutschland. Mindestlöhne in Europa.

[4] Butterwegge C (2018). Krise und Zukunft des Sozialstaates.

[5] Butterwegge C, Quenzel G, Hurrelmann K (2019). Bildung – ein probates Mittel zur Bekämpfung der (Kinder-)Armut in Deutschland? – Was getan werden muss, damit sich die Kluft zwischen Arm und Reich wieder schließt. Handbuch Bildungsarmut.

[6] Butterwegge C (2020). Die zerrissene Republik. Wirtschaftliche, soziale und politische Ungleichheit in Deutschland.

[7] Butterwegge C (2020). Ungleichheit in der Klassengesellschaft.

[8] Butterwegge C, Klundt M, Belke-Zeng M (2008). Kinderarmut in Ost- und Westdeutschland.

[9] Chassé KA, Zander M, Rasch K (2007). Meine Familie ist arm. Wie Kinder im Grundschulalter Armut erleben und bewältigen.

[10] Gintzel U, Clausnitzer S, Drößler T, Mummert L, Rudolph M (2008). Kinderarmut und kommunale Handlungsoptionen.

[11] Greiffenhagen S, Neller K (2005). Praxis ohne Theorie? Wissenschaftliche Diskurse zum Bund-Länder-Programm „Stadtteile mit besonderem Entwicklungsbedarf – die Soziale Stadt“.

[12] Hanesch W (2011). Die Zukunft der „Sozialen Stadt“. Strategien gegen soziale Spaltung und Armut in den Kommunen.

[13] Henschel A, Krüger R, Schmitt C, Stange W (2009). Jugendhilfe und Schule. Handbuch für eine gelingende Kooperation.

[14] Hübenthal M (2018). Soziale Konstruktionen von Kinderarmut. Sinngebungen zwischen Erziehung, Bildung, Geld und Rechten.

[15] Klundt M (2019). Gestohlenes Leben. Kinderarmut in Deutschland.

[16] Klundt M (2020). Krisengerechte Kinder statt kindergerechtem Krisenmanagement? Auswirkungen der Corona-Krise auf die Lebensbedingungen junger Menschen.

[17] Lutz R, Lutz R, Hammer V (2010). Verwirklichungskulturen als kommunale Armutsprävention. Wege aus der Kinderarmut. Gesellschaftspolitische Rahmenbedingungen und sozialpädagogische Handlungsansätze.

[18] März D (2017). Kinderarmut in Deutschland und die Gründe für ihre Unsichtbarkeit.

[19] Ottersbach M, Platte A, Rosen L (2016). Soziale Ungleichheiten als Herausforderung für inklusive Bildung.

[20] Pusch T (2018). Bilanz des Mindestlohns: deutliche Lohnerhöhungen, verringerte Armut, aber auch viele Umgehungen.

[21] Reich K (2014). Inklusive Didaktik. Bausteine für eine inklusive Schule.

[22] Schulten T, Weinkopf C, Körzell S, Falk C (2015). Die Einführung des gesetzlichen Mindestlohns – eine erste Zwischenbilanz. Kommt der Mindestlohn überall an? – Eine Zwischenbilanz.

[23] Steinmeier F-W (1992). Bürger ohne Obdach – Zwischen Pflicht zur Unterkunft und Recht auf Wohnraum. Tradition und Perspektiven staatlicher Intervention zur Verhinderung und Beseitigung von Obdachlosigkeit.

[25] Stolz-Willig B, Butterwegge C, Klundt M (2003). Generationen- und Geschlechtergerechtigkeit oder: Familienarbeit neu bewerten – aber wie?. Kinderarmut und Generationengerechtigkeit. Familien- und Sozialpolitik im demografischen Wandel.

[24] Walther U-J (2002). Soziale Stadt – Zwischenbilanzen. Ein Programm auf dem Weg zur Sozialen Stadt?.

[26] Zander M, Weiß H (2000). Kinderarmut und Existenzsicherung im Sozialstaat. Frühförderung mit Kindern und Familien in Armutslagen.

